# Genetic diversity and population structure of *Plasmodium falciparum *in Thailand, a low transmission country

**DOI:** 10.1186/1475-2875-8-155

**Published:** 2009-07-14

**Authors:** Tepanata Pumpaibool, Céline Arnathau, Patrick Durand, Naowarat Kanchanakhan, Napaporn Siripoon, Aree Suegorn, Chitr Sitthi-amorn, François Renaud, Pongchai Harnyuttanakorn

**Affiliations:** 1 Biomedical Science, Graduate School, Chulalongkorn University, Bangkok 10330, Thailand; 2 Malaria Research Programme, College of Public Health Science, Chulalongkorn University, Bangkok 10330, Thailand; 3 Génétique et Evolution des Maladies Infectieuses, Unité Mixte de Recherche-Institut de Recherche pour le Développement/Centre National de la Recherche Scientifique 2724, B.P. 64501,34394 Montpellier Cedex 5, France; 4 Department of Biology, Faculty of Science, Chulalongkorn University, Bangkok 10330, Thailand

## Abstract

**Background:**

The population structure of the causative agents of human malaria, *Plasmodium *sp., including the most serious agent *Plasmodium falciparum*, depends on the local epidemiological and demographic situations, such as the incidence of infected people, the vector transmission intensity and migration of inhabitants (i.e. exchange between sites). Analysing the structure of *P. falciparum *populations at a large scale, such as continents, or with markers that are subject to non-neutral selection, can lead to a masking and misunderstanding of the effective process of transmission. Thus, knowledge of the genetic structure and organization of *P. falciparum *populations in a particular area with neutral genetic markers is needed to understand which epidemiological factors should be targeted for disease control. Limited reports are available on the population genetic diversity and structure of *P. falciparum *in Thailand, and this is of particular concern at the Thai-Myanmar and Thai-Cambodian borders, where there is a reported high resistance to anti-malarial drugs, for example mefloquine, with little understanding of its potential gene flow.

**Methods:**

The diversity and genetic differentiation of *P. falciparum *populations were analysed using 12 polymorphic apparently neutral microsatellite loci distributed on eight of the 14 different chromosomes. Samples were collected from seven provinces in the western, eastern and southern parts of Thailand.

**Results:**

A strong difference in the nuclear genetic structure was observed between most of the assayed populations. The genetic diversity was comparable to the intermediate level observed in low *P. falciparum *transmission areas (average *H*_S _= 0.65 ± 0.17), where the lowest is observed in South America and the highest in Africa. However, uniquely the Yala province, had only a single multilocus genotype present in all samples, leading to a strong geographic differentiation when compared to the other Thai populations during this study. Comparison of the genetic structure of *P. falciparum *populations in Thailand with those in the French Guyana, Congo and Cameroon revealed a significant genetic differentiation between all of them, except the two African countries, whilst the genetic variability of *P*. *falciparum *amongst countries showed overlapping distributions.

**Conclusion:**

*Plasmodium falciparum *shows genetically structured populations across local areas of Thailand. Although Thailand is considered to be a low transmission area, a relatively high level of genetic diversity and no linkage disequilibrium was found in five of the studied areas, the exception being the Yala province (Southern peninsular Thailand), where a clonal population structure was revealed and in Kanchanaburi province (Western Thailand). This finding is particularly relevant in the context of malaria control, because it could help in understanding the special dynamics of parasite populations in areas with different histories of, and exposure to, drug regimens.

## Background

Malaria is still one of the most important infectious diseases, endemic in the tropical and sub-tropical parts of about 102 countries, especially in the African continent. In Thailand, although the total number of malaria cases has been decreasing annually [1], malaria remains most prevalent along the Thai borders with Myanmar, Cambodia and Malaysia, including for *Plasmodium falciparum*, the agent of the most malignant form of malaria that accounts globally for some 300–600 million cases of clinical malaria and 1.5 – 2.7 million deaths each year. Although *P. falciparum *can cause the most severe of the four human forms of malaria [2], the clinical manifestations are not always severe, but are somewhat pleomorphic ranging from asymptomatic parasitaemia (carriers) to potentially fatal cerebral infection and multiple organ failure, making the epidemiology more complicated. Moreover, Thailand is a known epicenter of *P. falciparum *drug resistance [3]. At present, *P. falciparum *strains have become more resistant, in terms of both resistance level and frequency in the population, to mefloquine, in addition to the widespread resistance to chloroquine, and sulphadoxine/pyrimethamine observed in many endemic areas, especially within those provinces located along the Thai-Myanmar border (i.e. Tak, Ranong and Kanchanaburi), or the Thai-Cambodian border (i.e. Trat, Chantaburi and Sa Kaeo). As a result of declining mefloquine efficacy, a combination of mefloquine and artesunate is now administered in those areas [4]. The *Plasmodium falciparum *gene flow between transmission areas and the existent level of *P. falciparum *population genetic diversity are directly implicated in the spread of drug resistance. Understanding the genetic complexity and organization (structure) of *P. falciparum *populations is a crucial aspect for the control of this disease, since the genetic diversity and population structure of *P. falciparum *in each location will have profound impacts on clonal diversity [5], competitive or synergistic interactions amongst clones [6-8], dynamics of drug resistance [9], persistence of the asexual infection and gametocyte production [10], infectivity in each relevant mosquito vectors [11] and malaria vaccine development [12].

Globally, *P. falciparum *is known to exhibit a diverse and patchy array of population genetic characteristics, which are apparently correlated with local levels of endemicity and transmission intensity [5]. However, the rapidly declining endemicity may lead to a more fragmented population structure with greater genetic isolation between endemic foci, whilst the decreased levels of gene flow may slow gene flow between populations and limit the spread of resistance between populations, but also enhance the rate of evolution of multiple resistance phenotypes [13]. It thus may become increasingly important to understand the fragmented nature genetic structure of residual parasite populations.

The *P. falciparum *transmission rate [14,15], and the migration of infected (carrier) human inhabitants [16], which differ in each endemic area, affect the genetic variation and population genetic structure of this parasite. For example, it is unclear whether parasites are commonly spread from one area to another by migrants or whether they emerge from local endemic populations. They will also be subjected to local host immunity, both in mosquitoes (species and local populations) and in their humans as hosts. Several studies have documented this relationship [16-18]. At a global scale, significant differences in the population structure of *P. falciparum *in different locations have been reported [5]. A strong linkage disequilibrium, low genetic diversity and high levels of geographic genetic differentiation were observed in countries with a low transmission intensity (i.e. South America and Southeast Asia) [5,19,20], whilst random gene recombination amongst loci, a high genetic diversity and low levels of geographical differentiation were observed in regions of Africa where transmission is high [5]. In contrast, a significant linkage disequilibrium with high genetic diversity was observed in the Republic of the Congo, an African region of high parasite transmission [21]. Moreover, high linkage disequilibrium of *P. falciparum *populations in Kenya was also observed where this form of malaria is also transmitted at a relatively high frequency [22]. Geographical variation in the extent of parasite inbreeding may have consequences for the success of potential malaria-control strategies. The degree of inbreeding modifies the effective recombination rate and so may affect the rate of increased drug resistance when more than one locus is involved. Higher inbreeding levels may allow a more rapid increase of multilocus drug resistant phenotypes [13,23]. However, recombination between genetically different clones has a potential to generate parasites exhibit a range of responses to different drugs [14,24].

Understanding of the genetic structure of *P. falciparum*, as well as other malaria parasites such as *Plasmodium vivax*, is essential for predicting how fast given phenotypes, such as drug or host resistance or novel antigenic variants, will originate and spread within and between populations [25]. Studies of the population structure at a local scale are more likely to be informative rather than misleading, and are clearly needed to understand the dynamics of *P. falciparum *populations, and to lead to an efficient management of this disease agent in particular areas. Indeed, the genetic composition and evolutionary change of each *P. falciparum *population is of great importance to ascertain the ecophysiology of the parasite and host-parasite interactions, as well as the evolution of resistance in the pathogen. Studies using the extensive polymorphism in antigen coding loci provide little valid information on the population structure of *P. falciparum *since, being under strong but varying selection, they reflect the combined effects of population history and natural selection [26].

In Thailand, most *P. falciparum *population genetic studies have focused on the boundary with Myanmar in the western part using different genetic markers [5,27,28], but the *P. falciparum *genetic structure relies on the epidemiological and demographical situations encountered in different areas and may change within a relatively short period of time. In this report, the population genetic structure of *P. falciparum*, is evaluated in seven provinces of Thailand, using 12 polymorphic and apparently neutral nuclear microsatellite loci as genetic markers. These were also compared with samples from other endemic countries, while using the same loci described published literature for other populations. Such studies could later be extended to other endemic areas displaying different treatment policies and different uses of prophylactic anti-malarial drugs.

## Methods

### Study sites

**Figure 1:**
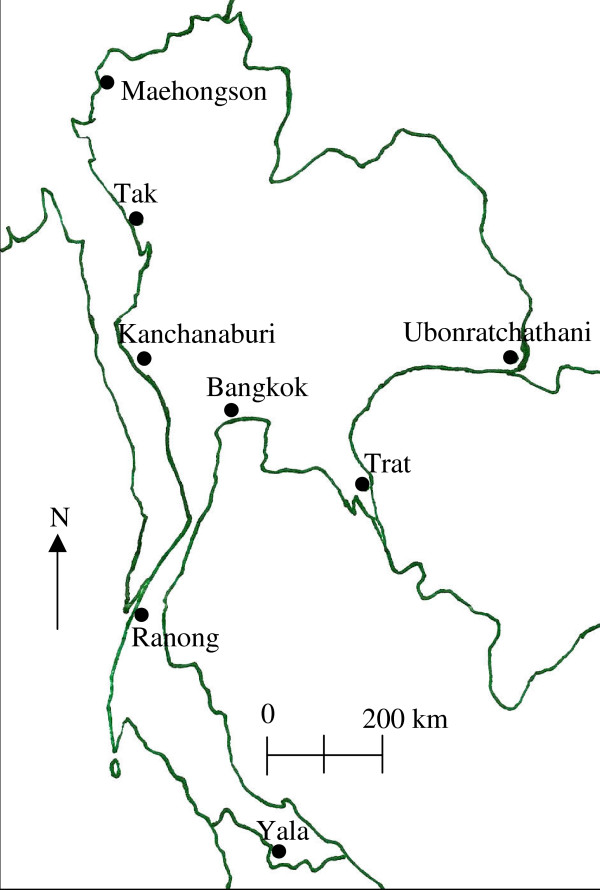
**Blood collection sites**. Localization of the seven provinces in Thailand from which *P falciparum *infected blood samples were obtained. Maehongson, Tak, Kanchanaburi and Ranong sites are located on the border with Myanmar. Ubonratchathani and Trat are located in the east along the border with Laos and Cambodia, respectively. Yala is a province nearby Malaysia.

The parasitized blood samples were collected from patients who attended for malarial diagnosis at clinics in seven provinces of Thailand from 2002 to 2007 (Figure 1). All patients had uncomplicated malaria. Five of these provinces (Tak, Yala, Maehongson, Kanchanaburi and Ranong) were ranked in the top ten provinces for the occurrence of *P. falciparum *based malaria incidences in Thailand [29]. In 2006, the malaria incidence in these provinces ranged from the highest in Tak, at 8,648 cases; through 3,544, 2,411, 1,250, 1,072 and 629 cases in Yala, Maehongson, Kanchanaburi, Ranong and Trat provinces, respectively. In addition, although there have been no cases reported in Ubonratchathani since 2005, in 2004 there were 979 cases reported in this province [29]. Four of these seven provinces, Maehongson, Tak, Kanchanaburi and Ranong, are located along the Thailand-Myanmar border in the west of Thailand, whilst Ubonratchathani and Trat locate along the Thailand-Laos and Thailand-Cambodia borders, respectively, in Eastern Thailand. Lastly, Yala is located in the southern part of Thailand, close to Malaysia (Figure 1). There is a relatively high exchange rate of people who move between international borders, especially between Thailand and Myanmar [4]. The samples were collected from Mueang and Mae Sot districts in the Maehongson and Tak provinces, respectively, where the highest malaria incidence was reported, whilst in Kanchanaburi and Ranong provinces, the blood sample collections were conducted in the Sai Yok and Mueang districts, respectively. Finally, in the Yala province, the sample collection was performed in the malaria clinic in the Bannang Sata district, where the malaria incidence has increased in frequency in relation to recent political violence in the three southernmost provinces (Yala, Narathiwat and Songkhla) since 2004. The malaria incidence in Yala increased annually between 2004 and 2006 at 1,903 to 3,544 cases. Because of the difficulty of access to this, only a single year of samples were obtained from Yala. In the eastern part, the blood samples from Na Chaluai and Buntharik districts in Ubonratchathani province and Bo Rai district in Trat province were obtained. These two provinces had a lower malaria incidence than that of the other five.

### *Plasmodium falciparum *isolates

The *P. falciparum*-infected human blood samples from two sources in Thailand were used in the study: 1) samples from *in vitro *culture, which had been preserved in liquid nitrogen at the Malaria Research Programme, College of Public Health Sciences, Chulalongkorn University, and 2) blood samples from *P. falciparum *malaria patients preserved on filter papers. Blood samples collected before 2004 were *in vitro *cultivated after blood collection from the malaria patient, and the cultivated samples were then cryopreserved in liquid nitrogen until being used in this study. The DNA was extracted from cultured parasites by standard phenol/chloroform extraction of whole blood. Thereafter, between 2004 and 2007, parasitized blood samples were collected from thick film-confirmed falciparum malaria patients, before drug administration. These patients were informed of the objectives of this study, agreed to participate in it, and signed a standard consent form. Blood samples were taken from finger-pricks of *P. falciparum-*infected (malaria symptomatic) patients, and were absorbed onto 3 MM Whatman filter paper and air-dried. DNA from the dried blood sample was subsequently extracted using a DNeasy Tissue kit (Qiagen) according to the manufacturer's instructions. The blood collection protocol was approved by the ethical committee of the Institute of Health Research, Chulalongkorn University. Socio-demographic information of patients was collected by interview, especially the most likely place of infection, to help identify the parasite's origin. The provincial origins and year of collection of the infected blood samples, and the likely country of infection source where not Thailand, are summarized in Table 1.

**Table 1:** Collected blood samples. *P. falciparum *infected blood samples; year and locality collected from and likely origin of the infection when not within Thailand.

	Number of samples collected in the year						Total number	
Province	2002	2003	2004	2005	2006	2007	Total	Outside Thailand
Maehongson	--	2	2	16	7	5	32	2 (6.3%)^a^
Tak	--	4	10	--	--	57	71	51 (72%)^a^
Kanchanaburi	15	--	63	61	77	50	266	37 (14%)^a^
Ubonratchathani	--	5	3	6	11	--	25	3 (12%)^b^
Trat	--	9	5	4	12	4	34	7 (21%)^c^
Ranong	--	10	10	9	13	17	59	43 (73%)^a^
Yala	--	--	--	--	--	36	36	0

Total	15	30	93	96	120	169	523	143 (27%)

The likely source of *P. falciparum *infection(s), when outside of the province the blood sample was collected from, are shown (Outside Thailand) as the total number and of all samples in that province (%) where ^a^, ^b ^and ^c ^represent Myanmar, P.D.R. of Laos and Cambodia, respectively.

### Microsatellite genotyping

For genotyping *P. falciparum *from each infected blood sample, the DNA was extracted and used as template for the PCR based amplification of 12 established polymorphic and apparently neutral microsatellite loci distributed on eight of the 14 different chromosomes [30], as listed in Table 2. Both a one-step PCR and two-step semi-nested PCR strategy with fluorescent end-labeled primers were used for the microsatellite amplification, as described in Razakandrainibe and colleagues [22], following the methodology developed by Anderson *et al *[30]. For comparison of the international samples and with the published literature, the first seven microsatellite markers listed in Table 2 were used, as well as for screening if the samples were single or multiple isolate infected (see below). All Thai samples were further screened with the other five loci. The size (length) of the amplified microsatellite repeats were resolved and sized relative to an internal size standard on an ABI Prism 310 Genetic Analyzer using Genescan software. Thus, and importantly for the analysis, size variation can be derived from indels in the flanking sequences as well as in changes in the microsatellite core repeat unit numbers, whilst substitution mutations in any part of the amplicon are overlooked.

**Table 2:** Microsatellites used in this study.

Microsatellite marker	Chromosome	GenBank access no.	Number of alleles	Size range (base pairs)
Poly α	4	L18785	17	131–189^a^
TA60	13	AF010556	11	64–94^b^
ARA2	11	X17484	10	51–81^b^
Pfg377	12	L04161	4	95–104^b^
PfPK2	12	X63648	9	160–193^b^
TA87	6	AF010571	11	78–116^b^
TA109	6	AF010508	7	157–185^a^
TA80	10	G38857	4	139–151
ARP2	13	G37793	7	160–184
TA1	6	AF010507	12	154–190^b^
TA81	5	AF010510	11	109–142^a^
C1M8	1	G38013	20	150–216

Characteristics of the 12 *P. falciparum *microsatellite loci with the marker code, chromosome location, GenBank accession number, the number of microsatellite alleles detected per locus and the size range of the amplified alleles in base pairs.

The primer sequences for each pair of PCR primers, and PCR conditions are as already reported [30]. Loci have been previously reported to comply with the IAM^a ^or SMM^b ^models of evolution and this suitable for *F*_ST _or *R*_ST _analysis, respectively.

### Data analysis

Only isolates that successfully amplified at all 12 loci within the Thai samples or at the selected seven loci for international samples were included in the respective analyses. Moreover, for population genetics, only those samples that showed a single *P. falciparum *clone infection were analysed, because of the difficulty in defining multi-locus genotypes in samples with a polyclonal infection. To this end, given that these 12 loci are all single genome copy, not in linkage, and that *P. falciparum *from the blood (schizonts and early trophozoites) has a haploid genome, then isolates with more than one allele at more than one locus were deemed to be from multiple infections. Genetic diversity was measured by the allelic richness per locus and sample (*R*_S_) and Nei's unbiased expected heterozygosity (*H*_S_), after adaption to haploid data, using FSTAT, v2.9.4 [31]. Differences in the allelic richness and expected heterozygosities were tested by an exact Wilcoxon rank-sum test using the SYSTAT software.

Linkage disequilibrium analyses between pairs of loci were performed with FSTAT v2.9.4. Genetic variation and differentiation were analyzed using FSTAT. Canonical correspondence analysis (CCA) was carried out to illustrate the measures of population structure, using CANOCO^® ^software [32,33]. Only isolates scored at each locus were considered for CCA. Analysis was performed with only the single infected samples. The significance of the canonical axes was tested with a Monte Carlo permutation test [32], which also allowed estimation of the 95% confidence intervals of the centroid of each population.

## Results

### Multiple isolate infections

Because the haploid stage of *P. falciparum*, i.e. schizonts and early trophozoites, present in human blood samples were analysed, the presence of multiple infections was assessed in a given isolate by the presence of more than one allele at any of the microsatellite locus surveyed. The minimal multiplicity of infection for each sample (i.e. the number of genetically distinct parasite genotypes in each sample) was analysed locus by locus, and was estimated from the locus that exhibited the highest number of alleles in a given isolate. Therefore, a single *P. falciparum *isolate infection is deemed to correspond to an isolate exhibiting only one allele at each of the 12 loci investigated. Of course, multiple infections that are genetically distinct overall but identical at the 12 microsatellite loci, for example recombinants or new mutations at other sites, or whose differences lead to null alleles and so would not be detected, would not be distinguished. Although the former would still be likely to be closely related isolates all the same, the later maybe quite different and thus the data represents the minimal rather than the actual level of multiple infections. Accordingly, the minimal proportion of multiple infections in each sample group was assessed for each locus and between loci in a sample group (Table 3). In the case of multiple infections, it is impossible to match the different alleles of each distinct locus to reconstruct a valid, rather than a chimeric (random) genotype, and so the analyses of the population structure must thus be conducted on single infected isolates only. In total, 523 blood samples from seven provinces of Thailand were genotyped at 12 microsatellite loci. Amongst these, 42 samples were unsuccessfully genotyped due to missing data at any loci, leaving 481 samples that were successfully genotyped at 12 polymorphic loci. Of these, 135 samples were found to be multiple *P. falciparum *isolate infected (28%), leaving 346 single isolate infected (72%) samples suitable for genotyping and population genetic analysis. The multiple infected samples were distributed across six of the seven provinces assayed, with frequencies of multiple infections as a ratio of infected individuals to the total number of samples ranging from 44% in Maehongson to 22% in the Ubonratchathani population (Table 3). An important result was that, in the Yala population, all 31 isolates analysed displayed an identical haplotype (i.e. genotype) across all 12 loci. However, since they were only sampled in one year this may reflect a transient expansion of a single clonal focal point, rather than a stable but isolated (inbred) population.

**Table 3:** Clonality characteristics.

Site	2002	2003	2004	2005	2006	2007	n	n*	Single^1^	Multiple^2^ No. (%)
Maehongson	-	2	2	16	7	5	32	32	18	14 (44%)
Tak	-	4	10	-	-	57	71	70	48	22 (31%)
Kanchanaburi	15	-	63	61	77	50	266	241	175	66 (27%)
Ubonratchathani	-	5	3	6	11	-	25	23	18	5 (22%)
Trat	-	9	5	4	12	4	34	28	18	10 (36%)
Ranong	-	10	10	9	13	17	59	56	38	18 (32%)
Yala	-	-	-	-	-	36	36	31	31	0 (0%)

Total	15	30	93	96	120	169	523	481	346	135 (28%)

Single and multiple *P. falciparum *isolate-infected blood samples, from seven provinces in Thailand, detected by PCR amplification of the12 polymorphic nuclear microsatellite markers.

n = number of collected isolates; n* = number of isolates successfully genotyped at all 12 microsatellite loci; The number of single^1 ^and multiple^2 ^(%) infected patient samples where multiple infected exhibit at least more than one allele at one locus.

### Population genetic analysis

For the population genetic analysis, based on 12 polymorphic nuclear microsatellite loci, only the 346 single *P. falciparum *isolate infected samples were considered. The number of alleles per locus varied from 4 to 20 alleles (Tables 2 and Additional file 1), and the allelic richness varied from 1.0 (Yala for all loci) to 10.1 (Tak for locus C1M8) (Table 4). When the average allelic richness per locus was compared amongst the seven Thai populations, Trat and Yala populations had a significantly lower allelic richness (Table 4). Gene diversity per microsatellite locus varied from 0.391 (locus TA109) to 0.841 (locus C1M8), whilst the gene diversity (*H*_S_) in each population was 0.68 ± 0.23, 0.63 ± 0.25, 0.60 ± 0.18, 0.70 ± 0.21, 0.56 ± 0.25, 0.62 ± 0.20 and 0 in the Maehongson, Tak, Kanchanaburi, Ubonratchathani, Trat, Ranong and Yala populations, respectively.

**Table 4:** Allelic richness.

	MAE	TAK	KAN	UBO	TRA	RAN	YAL
MAE	5.5 ± 2.3						
TAK	0.433	5.9 ± 2.4					
KAN	0.875	0.158	5.7 ± 2.2				
UBO	0.875	0.272	1.000	5.6 ± 2.5			
TRA	0.023*	0.002*	0.003*	0.008*	4.4 ± 1.9		
RAN	0.158	0.019*	0.099	0.480	0.158	5.0 ± 1.9	
YAL	0.002*	0.002*	0.002*	0.002*	0.002*	0.002*	1.0 ± 0.0

Mean allelic richness ± 1 S.D. in the diagonal. Wilcoxon Signed Ranks Test of allelic richness among populations with two-sided probabilities, using a normal distribution approximation, under the diagonal (* significant *p-value*). MAE, Maehongson; TAK, Tak; KAN, Kanchanaburi; UBO, Ubonratchathani; TRA, Trat; RAN, Ranong; YAL, Yala.

Linkage disequilibrium (LD) was tested at three levels, (i) for the whole population (ii) for the three regional areas; and (iii) amongst sites.

(i) For within the whole population (346 individuals), out of the 66 possible pairwise linkages, 34 significant (*p-value *= 0.00076) associations of loci were found.

(ii) For within the three regions, we found that in the Eastern region (Ubonratchathani and Trat provinces, n = 36), Western (Maehongson, Tak and Kanchanaburi provinces, n = 241) and Southern area, (Ranong and Yala provinces, n = 69), there were 0, 3 and 46 significant combinations of loci (out of 66) that were potentially in linkage (*p-value *= 0.00025), respectively.

(iii) For within all the sites, no significant (*p-value *= 0.00011) linkage disequilibrium was found in any combined pair of loci except for two combinations in Kanchanaburi (Polyα × TA81 and Ara2 × TA81), and, of course, a total linkage disequilibrium existed in the Yala population, since all samples have the same genotype.

Genetic differentiation (*F*_ST_) was estimated using the FSTAT software. A permutation test was applied (n = 10,000) permuting alleles amongst all samples for all loci to test whether *F*_ST _significantly differed from zero. A significant differentiation (*p-value *= 0.0024) of *P. falciparum *populations between sites was observed, with a very high differentiation between the Yala population and all the other populations (Table 5). The analysis of the genetic differentiation in *P. falciparum *populations between the three regions revealed a significant level of differentiation (i.e. *p-value *= 0.017) between the western, eastern and southern parts of Thailand with *F*_ST _values equal to (i) 0.0478 for the western versus the eastern regions, (ii) 0.1321 for the western versus the southern regions and (iii) 0.1199 for the eastern versus the southern regions.

**Table 5:** Genetic differentiation (*F*_ST_) between *P. falciparum *populations from seven provinces of Thailand.

Province	MAE (n = 18)	TAK (n = 48)	KAN (n = 175)	UBO (n = 18)	TRA (n = 18)	RAN (n = 38)	YAL (n = 31)
MAE	-	0.001	0.037**	0.015	0.048	0.012	0.657**
TAK		-	0.015**	0.038**	0.082**	0.020**	0.559**
KAN			-	0.050**	0.076**	0.012	0.474**
UBO				-	0.030	0.038**	0.619**
TRA					-	0.051**	0.693**
RAN						-	0.584**
YAL							-

**significant at *p-value *= 0.0024 after Bonferroni correction for multi tests.

MAE, Maehongson; TAK, Tak; KAN, Kanchanaburi; UBO, Ubonratchathani; TRA, Trat; RAN, Ranong; YAL, Yala, n = number of individuals.

### Genotype distribution

Three hundred different genotypes were obtained from the 346 single *P. falciparum *isolate infected individuals, with 14 identical haplotypes (Table 6). Thus, essentially identical genotypes were found from patients:

**Table 6:** Spatial and temporal genotype distribution of *P. falciparum *in Thailand.

Site	Number of individuals	different haplotype	identical haplotype in 2 individuals	identical haplotype in 3 individuals	identical haplotype in 7 individuals	identical haplotype in 31 individuals
Maehongson	18	18	-	-	-	-
Tak	48	47	1 in 2007	-	-	-
Kanchanaburi	175	162	-	1 in 2004	-	-
			2 in 2005	-	-	-
			1 in 2006	-	-	-
			3 in 2007	-	1 in 2007	-
Ubonratchathani	18	17	1 in 2003	-	-	-
Trat	18	18	-	-	-	-
Ranong	38	35	1 in 2005 and 2006	-	-	-
			1 in 2006 and 2007	-	-	-
			1 in 2007	-	-	-
Yala	31	-	-	-	-	1 in 2007

- in the same year but infected in two different areas: In Kanchanaburi, an identical genotype was found in seven individuals infected in 2007 where four of them were infected in Myanmar and three of them infected in Thailand,

- in the same site but collected in two successive years: In Ranong, an identical genotype was found in two individuals infected in 2005 and 2006, and another identical genotype was found in 2006 and 2007,

- in Yala, only one haplotype was found in 31 parasite samples.

These genotypes which are found in more than one individual would have an impact on the epidemiology of the disease because, (i) they can pass through several generations, and (ii) these genotypes could carry important traits, such as drug-resistance genes.

### Comparison to other *P. falciparum *blood samples from Africa and South America

**Figure 2:**
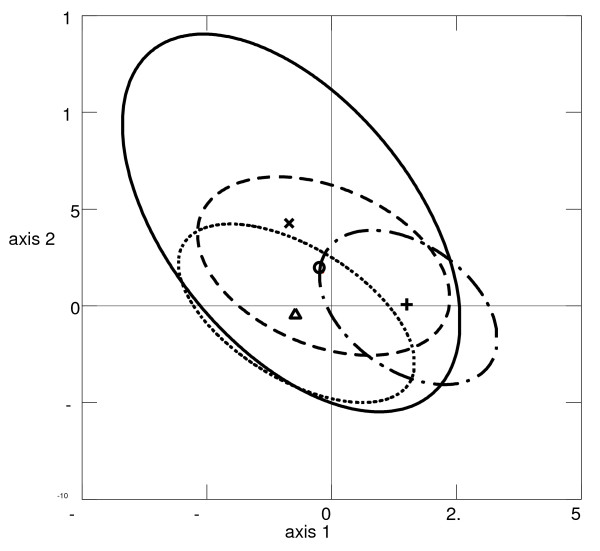
**Results of canonical correspondence analysis (CCA)**. Relative contribution of the variables "sites" and "genotypes" to the genetic structure of *P. falciparum *populations in four sites. *P. falciparum *populations were projected on the first two axes of the CCA. Centroids (dots) of each parasite populations are surrounded by the 95% C.I.s (ellipses). (**x **and solid oval stand for Congo population (n = 15), **o **and dash oval stand for the Cameroon population (n = 35), **+ **and long dash dot oval stand for the French Guyana population (n = 137) and Δ and round dot oval stand for the Thailand population (n = 286).

In order to compare the population genetics and structure of the Thai *P. falciparum *populations with the already published reports for other populations, the observed patterns at seven common microsatellite loci (Poly α, TA60, ARA2, Pfg377, PfPK2, TA87 and TA109), were compared with those observed from blood samples of two African countries, Congo (n = 15) and the Cameroon (n = 35), and one South American country, French Guyana (n = 137) (unpublished data). The gene diversity in the Thai populations (*H*_S _= 0.65 ± 0.17) was lower than those found in the two African countries (*H*_S _= 0.81 ± 0.07, and 0.78 ± 0.18 for the Congo and Cameroon, respectively), but higher than that of French Guyana (*H*_S _= 0.54 ± 0.08). Significant LD was observed in the two low transmission countries (12 and 6 out of 21 combinations for Thailand and French Guyana populations, respectively). The high LD value observed in Thailand is due to the Yala sample. The canonical correspondence analysis (CCA), searches for multivariate relationships between two data sets (e.g., genetic data of *P. falciparum *and countries environmental data) [33], was also used to determine the relative contribution of the countries (i.e. sites) to the global genetic structure of *P. falciparum *populations. A graphic representation of the results showed overlapping distributions (i.e. centroids and ellipses of the 95% CI – Figure 2) of the genetic variability of *P. falciparum *observed amongst sites. A Monte Carlo permutation test on the first four canonical correspondence analysis axes that combines parasite genotypes and sites is statistically significant (*p-value *= 0.001). This suggests some degree of geographic differentiation. Indeed, all populations in different countries displayed significant genetic differentiation except between the two African countries, Congo and Cameroon (Figure 2).

## Discussion

### Comparison of Thai *P. falciparum *genetic structure with other countries

At the level of continents, the genetic structure of *P. falciparum *populations in different continents revealed that African countries with high transmission intensities had the highest population genetic diversity. The genetic diversity decreased with low transmission intensities, as in Thailand (Asia) and French Guyana (South America). The average genetic diversity of *P. falciparum *populations in Thailand (*H*_S _= 0.65 ± 0.17) was, however, close to that of the high transmission intensity level of Papua New Guinea [5], and slightly higher than that reported for populations in Malaysia [19] and Brazil [20], also based on assaying genetic diversity at these seven microsatellite loci.

The deduced mating patterns of *P. falciparum *in Thailand showed a deviation from random mating, in congruence with the French Guyana population, whilst, in contrast, the two African populations showed likely panmixia. The proportion of multiple isolate infections, and the number of clones in an individual host, which are related to the transmission intensity of malaria parasites in any endemic area, are important parameters that affect *P. falciparum *mating, because fertilization may occur between gametes of the same or different genotypes in mosquitoes. Therefore, an inverse correlation between the proportion of multiple clone infections and the degree of linkage disequilibrium is expected. Even though the genetic diversity of Thai populations was observed at an intermediate level, and multiple isolate infections occurred frequently, a significant linkage disequilibrium was found which could be caused by, (i) a higher chance of inbreeding (selfing) in this parasite population, or (ii) structure formation within populations into sub-populations of each foci in Thailand (i.e. the Wahlund effect). The latter reason would be more likely to explain this finding because samples from all the foci from across the country (the seven different provinces) were pooled in the analysis.

### The genetic structure observed in each Thai locality

In Thailand, the malaria incidence rate shows a high spatial heterogeneity across the country, with high incidence regions found nearby the borders with Myanmar and Cambodia [34]. This study reports the genetic structure of *P. falciparum *analysis with 12 polymorphic nuclear microsatellite loci in isolates from multiple populations in seven provinces of Thailand.

The *P. falciparum *populations in the areas bordering with Myanmar revealed a similar level of genetic diversity (0.60 <*H*_S _<0.68), whilst the populations bordering with Laos and Cambodia had slightly higher or lower levels of genetic diversity at 0.70 and 0.56 in Ubonratchathani and Trat, respectively. Although the multiple isolate infection rates in those areas bordering with Myanmar (mean, 34%) were higher than that of Ubonratchanthani (22%) and comparable to that in Trat (36%), this could be explained by the notion that (i) the parasite mating in those areas is preferentially inbreeding, or (ii) the relatively small amount of sampled individuals in Trat and Ubonratchathani leading to a stochastic masking of the true population structure. The Yala population, however, showed an extreme difference in the gene diversity compared the other samples since it showed only a unique haplotype in all sampled individuals, but again may be a transient clonal expansion of an immigrant infection.

In contrast to the Yala population, a low but significant population structure was observed amongst the provinces that border with Myanmar, Laos and Cambodia. In the case of the Myanmar bounding provinces, the significant genetic structure detected between populations (KAN, TAK, MAE and RAN) was likely to be due to the fact that the pattern of human movement from neighbouring countries into each of these four provinces was different. In the Tak and Ranong provinces, the numerous movements in to Thailand from Myanmar from immigrant laborers, and from patients resident in Myanmar who cross the border to seek free malaria treatment, account for the high proportion (approximately 70%) of sampled individuals being likely to have been infected in Myanmar. Indeed, the same genotype was found in seven samples collected in 2007 in Kanchanaburi, from patients infected in Thailand and Myanmar. These, imported cases may play an important role on the *P. falciparum *gene pool in each endemic area and affect the epidemiology of the *P. falciparum *caused malaria in these areas. Besides this migration rate of humans in each foci, there is additionally the factor of the geographic barrier between Tak and Kanchanaburi provinces which may be another cause of genetic differentiation by partial isolation by distance and breaking down of panmixis. Thus, the genetic differentiation found between populations within each province of Thailand may reflect both a variation of the transmission rate in each location and the migration rate of people from one location to another. Certainly seasonal migration has been suspected as a leading cause of malaria transmission in these areas [35]. However, not only does the transmission rate and immigration of inhabitants play a role, but the differential dispersal of the *Anopheles *vectors in local areas may also have an effect on the parasite structure. In Thailand, *Anopheles aconitus sensu lato, Anopheles baimaii, Anopheles dirus, Anopheles maculatus, Anopheles minimus *and *Anopheles pseudowillmori *have been incriminated as important vectors of *P. falciparum *in humans, and they are differentially distributed throughout the country [36-38], and may each have different vectoring capabilities for different isolates of *P. falciparum*. Thus, although differences in the population structure of *P. falciparum *between two vectors (*Anopheles gambiae *and *Anopheles funestus*) have been preliminarily investigated [39], no such information currently exists for the different vector species in each region of Thailand.

Importantly, the same multilocus genotypes of *P. falciparum *infected blood samples collected one year apart (2005 – 2006 and 2006 – 2007) was observed only in Ranong, where one case was found in a patient infected from Myanmar and the following year was found from a patient infected in Thailand. If a generation time is assumed to be two months, this parasite genotype has been transmitted through six generations without detectable change across the 12 loci (eight/14 chromosomes) due to recombination. This may imply that if this *P. falciparum *strain has an advantage (fitness) in that particular environment, it will propagate and expand in this population.

### The genotype distribution of *P. falciparum *in Thailand and its consequence

In this study, both a spatial and a temporal distribution of *P. falciparum *haplotypes collected in the same year and over two years were detected. A unique spatial haplotype, distinct from the other populations, was found in all patients in the Yala population, which were obtained from individual residents in four different sub-districts in Bannang Sata and other three nearby districts during May – July 2007. Three possible explanations are available to explain this trend. Firstly, that a *P. falciparum *isolate with this genotype had a high infectivity in the mosquito's midgut or carried some genetic characteristics, such as drug resistance, which gave it a significant benefit over other haplotypes. Secondly, that there is no inhabitant movement into this area due to the political violence, and so as a result no new haplotype parasite was introduced into this area (no gene flow). Thirdly, there maybe a rare new gene influx by immigration but which was subsequently lost due to selection or stochastic failure to progress to a stable infection threshold. Finally, is the possibility that the decrease in sanitary cover in this area allowed this particular genotype to spread into the population rapidly. Thus, a longitudinal study in the population dynamics in this area is required. Nevertheless, this is the first study to reveal the potential clonal expansion of a single haplotype in a malaria endemic area.

## Conclusion

*Plasmodium falciparum *populations are highly structured in Thailand suggesting local and differential routes of genetic evolution for this parasite. Although Thailand is considered as a low transmission area, a high level of genetic diversity and weak linkage disequilibrium were found in most sites, except the transient infections in the Yala province in one year where only a single genotype was found. Moreover, (i) identical genotypes were encountered in the same site but from patients coming from different localities (i.e. Myanmar and Thailand), and (ii) two identical genotypes were observed from two successive years at the same site (i.e. Ranong); these findings are particularly relevant in the context of malaria control. They would help to understand the specific dynamics of malaria and parasite populations in areas displaying a different history of drug applications.

## Competing interests

The authors declare that they have no competing interests.

## Authors' contributions

TP carried out field work, the molecular genetic work, analysis, interpretation of data, and drafted the manuscript. CA carried out the molecular genetic work and data analysis. NK contributed to field work, DNA extraction of cultivated parasite. AS and NS were responsible for parasite cultivation. PD and FR carried out the statistic analysis, guidance for interpretation of data, participated in the manuscript preparation and revision. CS participated in the design of the study, the manuscript preparation and revision. PH participated in the design of the study, the manuscript preparation and revision and coordination. All authors read and approved the final manuscript.

## Supplementary Material

Additional file 1 **Allele frequencies**. Allele frequencies at 12 microsatellite loci in *P. falciparum *from seven Thai populations. Click here for file
