# Supplementary material for: Genetic diversity and population structure of Plasmodium falciparum in Thailand, a low transmission country
Source: Malar J. 2009 Jul 14;8:155. doi: 10.1186/1475-2875-8-155 (PMC2722663; doi:10.1186/1475-2875-8-155)
Supplement: Additional file 1 — Allele frequencies. Allele frequencies at 12 microsatellite loci in P. falciparum from seven Thai populations. [file 1475-2875-8-155-S1.doc]

**Allele frequencies.** Allele frequencies at 12 microsatellite loci in *P. falciparum* from seven Thai populations**.**

| Locus | allele size | MAE | TAK | KAN | UBO | TRA | RAN | YAL |
| --- | --- | --- | --- | --- | --- | --- | --- | --- |
| TA60 |  |  |  |  |  |  |  |  |
|  | n | 18 | 48 | 175 | 18 | 18 | 38 | 31 |
|  | 64 |  |  |  | 0.056 |  |  |  |
|  | 67 |  | 0.021 |  |  |  |  |  |
|  | 70 |  |  | 0.017 |  |  |  |  |
|  | 73 | 0.056 | 0.042 | 0.006 |  |  |  |  |
|  | 76 | 0.111 | 0.229 | 0.12 | 0.111 | 0.056 | 0.053 |  |
|  | 79 | 0.278 | 0.188 | 0.229 | 0.222 | 0.111 | 0.158 | 1.000 |
|  | 82 | 0.333 | 0.313 | 0.469 | 0.611 | 0.778 | 0.500 |  |
|  | 85 | 0.111 | 0.083 | 0.051 |  |  | 0.105 |  |
|  | 88 | 0.056 | 0.125 | 0.069 |  |  | 0.053 |  |
|  | 91 |  |  | 0.006 |  | 0.056 |  |  |
|  | 94 | 0.056 |  | 0.034 |  |  | 0.132 |  |

| Locus | allele size | MAE | TAK | KAN | UBO | TRA | RAN | YAL |
| --- | --- | --- | --- | --- | --- | --- | --- | --- |
| ARA2 |  |  |  |  |  |  |  |  |
|  | n | 18 | 48 | 174 | 18 | 18 | 38 | 31 |
|  | 51 |  |  | 0.006 |  |  |  |  |
|  | 57 | 0.111 | 0.042 |  |  |  |  |  |
|  | 60 |  |  |  | 0.056 |  | 0.026 |  |
|  | 63 | 0.111 | 0.042 | 0.023 | 0.222 | 0.167 | 0.026 |  |
|  | 66 | 0.278 | 0.333 | 0.247 | 0.222 | 0.389 | 0.237 |  |
|  | 69 |  | 0.063 | 0.080 |  |  | 0.053 |  |
|  | 72 | 0.333 | 0.354 | 0.511 | 0.278 | 0.389 | 0.526 |  |
|  | 75 | 0.111 | 0.125 | 0.086 | 0.222 | 0.056 | 0.053 | 1.000 |
|  | 78 |  | 0.042 | 0.046 |  |  | 0.079 |  |
|  | 81 | 0.056 |  |  |  |  |  |  |

| Locus | allele size | MAE | TAK | KAN | UBO | TRA | RAN | YAL |
| --- | --- | --- | --- | --- | --- | --- | --- | --- |
| TA87 |  |  |  |  |  |  |  |  |
|  | n | 18 | 48 | 174 | 18 | 18 | 38 | 31 |
|  | 78 |  |  |  |  | 0.056 |  |  |
|  | 86 | 0.056 | 0.042 |  |  |  |  |  |
|  | 89 | 0.056 | 0.042 | 0.006 |  |  |  |  |
|  | 92 |  |  |  | 0.056 |  |  |  |
|  | 95 |  | 0.021 | 0.029 | 0.278 |  | 0.053 |  |
|  | 98 | 0.222 | 0.125 | 0.103 | 0.056 | 0.056 | 0.132 |  |
|  | 101 | 0.111 | 0.188 | 0.126 | 0.222 | 0.222 | 0.289 |  |
|  | 104 | 0.222 | 0.104 | 0.063 | 0.167 |  | 0.132 |  |
|  | 107 | 0.222 | 0.438 | 0.615 | 0.167 | 0.333 | 0.368 | 1.000 |
|  | 110 | 0.111 | 0.042 | 0.011 | 0.056 | 0.278 |  |  |
|  | 116 |  |  | 0.046 |  | 0.056 | 0.026 |  |

| Locus | allele size | MAE | TAK | KAN | UBO | TRA | RAN | YAL |
| --- | --- | --- | --- | --- | --- | --- | --- | --- |
| Polyα |  |  |  |  |  |  |  |  |
|  | n | 18 | 48 | 175 | 18 | 18 | 38 | 31 |
|  | 131 |  |  | 0.011 |  |  |  |  |
|  | 136 |  |  | 0.011 | 0.056 |  |  |  |
|  | 142 | 0.056 |  |  |  |  |  |  |
|  | 146 | 0.111 | 0.229 | 0.400 | 0.111 | 0.278 | 0.211 |  |
|  | 150 | 0.111 |  | 0.034 | 0.056 |  | 0.026 |  |
|  | 153 |  | 0.021 | 0.006 |  | 0.056 | 0.053 |  |
|  | 156 | 0.167 | 0.208 | 0.251 | 0.278 | 0.333 | 0.211 |  |
|  | 160 | 0.111 | 0.104 | 0.069 | 0.222 |  | 0.079 |  |
|  | 163 | 0.056 |  | 0.023 |  |  |  |  |
|  | 166 |  | 0.021 | 0.006 | 0.056 |  |  |  |
|  | 169 | 0.056 | 0.042 | 0.051 | 0.111 | 0.111 | 0.105 |  |
|  | 172 | 0.222 | 0.208 | 0.069 | 0.111 | 0.056 | 0.158 |  |
|  | 175 |  | 0.021 | 0.017 |  |  |  |  |
|  | 178 |  | 0.042 | 0.029 |  | 0.056 | 0.026 |  |
|  | 181 | 0.056 |  |  |  |  |  |  |
|  | 183 |  | 0.083 | 0.011 |  |  |  |  |
|  | 189 | 0.056 | 0.021 | 0.011 |  | 0.111 | 0.132 | 1.000 |

| Locus | allele size | MAE | TAK | KAN | UBO | TRA | RAN | YAL |
| --- | --- | --- | --- | --- | --- | --- | --- | --- |
| Pfg377 |  |  |  |  |  |  |  |  |
|  | n | 18 | 47 | 175 | 18 | 18 | 38 | 31 |
|  | 95 | 0.056 | 0.106 | 0.080 |  |  | 0.053 |  |
|  | 98 | 0.833 | 0.851 | 0.800 | 0.611 | 0.500 | 0.737 |  |
|  | 101 | 0.111 | 0.043 | 0.114 | 0.389 | 0.500 | 0.184 | 1.000 |
|  | 104 |  |  | 0.006 |  |  | 0.026 |  |

| Locus | allele size | MAE | TAK | KAN | UBO | TRA | RAN | YAL |
| --- | --- | --- | --- | --- | --- | --- | --- | --- |
| PfPK2 |  |  |  |  |  |  |  |  |
|  | n | 18 | 48 | 173 | 18 | 18 | 38 | 31 |
|  | 160 |  |  |  | 0.056 |  |  |  |
|  | 163 | 0.111 | 0.042 | 0.139 | 0.056 | 0.389 | 0.158 |  |
|  | 166 | 0.444 | 0.396 | 0.156 | 0.167 | 0.333 | 0.263 |  |
|  | 169 | 0.111 | 0.083 | 0.243 | 0.333 | 0.167 | 0.211 |  |
|  | 172 | 0.167 | 0.313 | 0.399 | 0.278 | 0.111 | 0.263 | 1.000 |
|  | 175 | 0.167 | 0.063 | 0.006 | 0.056 |  | 0.026 |  |
|  | 178 |  |  | 0.029 |  |  | 0.026 |  |
|  | 184 |  | 0.042 |  |  |  |  |  |
|  | 193 |  | 0.063 | 0.029 | 0.056 |  | 0.053 |  |

| Locus | allele size | MAE | TAK | KAN | UBO | TRA | RAN | YAL |
| --- | --- | --- | --- | --- | --- | --- | --- | --- |
| TA109 |  |  |  |  |  |  |  |  |
|  | n | 18 | 48 | 174 | 18 | 18 | 38 | 31 |
|  | 157 |  |  | 0.069 |  |  | 0.053 |  |
|  | 160 | 0.778 | 0.917 | 0.822 | 0.889 | 0.889 | 0.789 |  |
|  | 163 |  | 0.021 | 0.017 |  | 0.056 |  |  |
|  | 170 |  |  | 0.006 | 0.056 |  |  |  |
|  | 173 | 0.222 | 0.042 | 0.08 |  |  | 0.158 | 1.000 |
|  | 176 |  | 0.021 |  | 0.056 | 0.056 |  |  |
|  | 185 |  |  | 0.006 |  |  |  |  |

| Locus | allele size | MAE | TAK | KAN | UBO | TRA | RAN | YAL |
| --- | --- | --- | --- | --- | --- | --- | --- | --- |
| TA80 |  |  |  |  |  |  |  |  |
|  | n | 17 | 48 | 150 | 18 | 18 | 38 | 31 |
|  | 139 |  |  | 0.007 |  |  |  |  |
|  | 145 | 0.294 | 0.521 | 0.527 | 0.333 | 0.111 | 0.500 |  |
|  | 148 | 0.647 | 0.417 | 0.427 | 0.667 | 0.889 | 0.421 | 1.000 |
|  | 151 | 0.059 | 0.063 | 0.040 |  |  | 0.079 |  |

| Locus | allele size | MAE | TAK | KAN | UBO | TRA | RAN | YAL |
| --- | --- | --- | --- | --- | --- | --- | --- | --- |
| ARP2 |  |  |  |  |  |  |  |  |
|  | n | 18 | 48 | 170 | 17 | 18 | 38 | 31 |
|  | 160 |  | 0.021 |  |  |  |  |  |
|  | 169 | 0.056 | 0.042 | 0.006 | 0.118 |  |  |  |
|  | 172 | 0.833 | 0.833 | 0.800 | 0.471 | 0.889 | 0.868 |  |
|  | 175 |  | 0.083 | 0.012 |  |  |  |  |
|  | 178 | 0.056 | 0.021 | 0.141 | 0.118 | 0.056 | 0.132 |  |
|  | 181 |  |  | 0.024 | 0.235 |  |  | 1.000 |
|  | 184 | 0.056 |  | 0.018 | 0.059 | 0.056 |  |  |

| Locus | allele size | MAE | TAK | KAN | UBO | TRA | RAN | YAL |
| --- | --- | --- | --- | --- | --- | --- | --- | --- |
| TA1 |  |  |  |  |  |  |  |  |
|  | n | 18 | 47 | 174 | 18 | 18 | 38 | 30 |
|  | 154 |  | 0.021 |  |  |  |  |  |
|  | 157 | 0.056 | 0.064 | 0.011 | 0.056 | 0.056 |  |  |
|  | 160 |  |  | 0.006 |  |  |  |  |
|  | 163 | 0.167 | 0.191 | 0.121 | 0.111 | 0.167 | 0.105 |  |
|  | 166 | 0.444 | 0.511 | 0.621 | 0.556 | 0.667 | 0.684 | 1.000 |
|  | 169 | 0.167 | 0.021 | 0.011 | 0.056 |  |  |  |
|  | 172 | 0.167 | 0.128 | 0.080 |  |  |  |  |
|  | 175 |  | 0.021 | 0.006 | 0.111 |  |  |  |
|  | 178 |  |  |  | 0.056 | 0.056 |  |  |
|  | 181 |  | 0.043 | 0.121 |  |  | 0.132 |  |
|  | 187 |  |  | 0.006 |  |  |  |  |
|  | 190 |  |  | 0.017 | 0.056 | 0.056 | 0.079 |  |

| Locus | allele size | MAE | TAK | KAN | UBO | TRA | RAN | YAL |
| --- | --- | --- | --- | --- | --- | --- | --- | --- |
| C1M8 |  |  |  |  |  |  |  |  |
|  | n | 18 | 47 | 153 | 17 | 17 | 37 | 31 |
|  | 150 |  |  |  | 0.059 |  |  |  |
|  | 156 |  | 0.021 | 0.013 |  | 0.059 |  |  |
|  | 159 | 0.167 | 0.043 | 0.150 | 0.118 | 0.471 | 0.270 |  |
|  | 162 | 0.167 | 0.064 | 0.039 | 0.176 | 0.059 | 0.054 |  |
|  | 165 |  |  | 0.013 |  |  |  |  |
|  | 168 | 0.056 | 0.170 | 0.131 | 0.118 | 0.059 | 0.027 | 1.000 |
|  | 171 | 0.111 |  | 0.013 | 0.059 |  | 0.054 |  |
|  | 174 | 0.056 | 0.106 | 0.007 | 0.118 | 0.118 | 0.027 |  |
|  | 177 | 0.278 | 0.340 | 0.34 | 0.118 | 0.118 | 0.405 |  |
|  | 180 |  | 0.021 | 0.02 | 0.059 |  |  |  |
|  | 183 | 0.167 | 0.064 | 0.072 |  |  | 0.162 |  |
|  | 189 |  |  | 0.013 | 0.118 |  |  |  |
|  | 192 |  | 0.064 | 0.124 |  |  |  |  |
|  | 195 |  | 0.043 | 0.007 | 0.059 |  |  |  |
|  | 198 |  | 0.043 | 0.007 |  |  |  |  |
|  | 201 |  |  | 0.007 |  |  |  |  |
|  | 204 |  |  | 0.007 |  |  |  |  |
|  | 207 |  | 0.021 | 0.026 |  | 0.059 |  |  |
|  | 210 |  |  | 0.013 |  |  |  |  |
|  | 216 |  |  |  |  | 0.059 |  |  |

| Locus | allele size | MAE | TAK | KAN | UBO | TRA | RAN | YAL |
| --- | --- | --- | --- | --- | --- | --- | --- | --- |
| TA81 |  |  |  |  |  |  |  |  |
|  | n | 18 | 44 | 172 | 17 | 17 | 37 | 29 |
|  | 109 | 0.056 |  | 0.023 |  |  | 0.027 |  |
|  | 112 | 0.056 | 0.068 | 0.076 | 0.059 | 0.059 | 0.054 |  |
|  | 115 | 0.167 | 0.386 | 0.384 | 0.353 | 0.176 | 0.243 |  |
|  | 118 | 0.167 | 0.159 | 0.035 | 0.118 |  | 0.054 |  |
|  | 121 | 0.222 | 0.159 | 0.233 | 0.118 | 0.235 | 0.486 |  |
|  | 124 | 0.278 | 0.114 | 0.134 | 0.118 | 0.176 | 0.054 |  |
|  | 127 |  | 0.091 | 0.064 | 0.176 | 0.353 | 0.081 | 1.000 |
|  | 130 | 0.056 |  |  | 0.059 |  |  |  |
|  | 133 |  | 0.023 | 0.017 |  |  |  |  |
|  | 139 |  |  | 0.023 |  |  |  |  |
|  | 142 |  |  | 0.012 |  |  |  |  |

MAE, Maehongson; TAK, Tak; KAN, Kanchanaburi; UBO, Ubonratchathani;

TRA, Trat; RAN, Ranong; YAL, Yala. n = number of samples analysed in each locality.
